# Risk of depression during pregnancy in usual risk antenatal care*

**DOI:** 10.1590/1518-8345.6463.3963

**Published:** 2023-07-21

**Authors:** Mônica Maria de Jesus Silva, Tainah Barbosa de Moraes Serrano, Giovanna da Silva Porcel, Bruna Borlina Monteiro, Maria José Clapis

**Affiliations:** 1 Universidade de São Paulo, Escola de Enfermagem de Ribeirão Preto, Centro Colaborador de la OPS/OMS para el Desarrollo de la Investigación en Enfermería, Ribeirão Preto, SP, Brasil.

**Keywords:** Depression, Pregnancy, Prenatal Care, Primary Health Care, Nursing, Obstetric Nursing, Depresión, Embarazo, Atención Prenatal, Atención Primaria de Salud, Nursing, Enfermería Obstétrica, Depressão, Gravidez, Cuidado Pré-Natal, Atenção Primária à Saúde, Enfermagem, Enfermagem Obstétrica

## Abstract

**Objective:**

to identify the risk of depression during pregnancy among pregnant women receiving routine prenatal care and the associated factors.

**Method:**

a cross-sectional study, carried out with 201 pregnant women, in a routine prenatal clinic of a university maternity hospital. Data were collected using an electronic form containing a characterization instrument and the *Escala de Risco de Depressão na Gravidez* (Depression during Pregnancy Scale). The dependent variable was the risk of depression during pregnancy. Statistical analysis was performed by calculating the Odds Ratio and using the Chi-square and Fischer’s Exact tests.

**Results:**

among the participants, 68.2% had a higher risk of depression during pregnancy. There was a statistically significant association between a higher risk of depression during pregnancy and occupation (p=0.04), that is, unemployment (OR=2.00) doubled the risk of depression.

**Conclusion:**

the high prevalence of the risk of depression during pregnancy indicates the necessity of planning, prioritizing, and integrating mental health into prenatal health services, especially in the primary healthcare environment, by health managers and policymakers.

## Abstract (es)

**Objetivo:**

identificar el riesgo de depresión durante el embarazo en gestantes de riesgo habitual incluidas en el control prenatal y los factores asociados.

**Método:**

estudio transversal, realizado con 201 gestantes, en el consultorio de prenatal de riesgo habitual de una maternidad universitaria. Para la recolección de datos se utilizó un formulario electrónico que contenía un instrumento de caracterización y la Escala de Riesgo de Depresión del Embarazo. La variable dependiente fue el riesgo de depresión en el embarazo. El análisis estadístico se realizó mediante el cálculo de la razón de posibilidades (*Odds Ratio*) y utilizando las pruebas de Chi-cuadrado y exacta de Fischer.

**Resultados:**

entre las participantes, 68,2% tenían mayor riesgo de depresión durante el embarazo. Hubo asociación estadísticamente significativa entre mayor riesgo de depresión durante el embarazo y la variable ocupación (p=0,04), o sea, la ausencia del trabajo (OR = 2,00) duplicó la probabilidad de ocurrencia.

**Conclusión:**

la alta prevalencia de riesgo de depresión durante el embarazo destaca la necesidad de planificación, priorización e integración de la salud mental en los servicios de salud prenatal, especialmente en el ámbito de la Atención Primaria de Salud, por parte de los gestores de salud y de los formuladores de políticas.

## Abstract (pt)

**Objetivo:**

identificar o risco de depressão na gravidez entre gestantes inseridas na assistência pré-natal de risco habitual e os fatores associados.

**Método:**

estudo transversal, realizado com 201 gestantes, no ambulatório de pré-natal de risco habitual de uma maternidade universitária. A coleta de dados utilizou um formulário eletrônico contendo um instrumento de caracterização e a Escala de Risco de Depressão na Gravidez. A variável dependente foi o risco de depressão na gravidez. A análise estatística deu-se pelo cálculo da razão de chances (*Odds Ratio*) e pelos testes Qui-quadrado e Exato de Fischer.

**Resultados:**

entre as participantes, 68,2% apresentaram maior risco de depressão na gravidez. Houve associação estatisticamente significativa entre o maior risco de depressão na gravidez e a variável ocupação (p=0,04), ou seja, a ausência de emprego (OR = 2,00) aumentou em duas vezes a chance de ocorrência.

**Conclusão:**

a alta prevalência de risco de depressão na gravidez evidencia a necessidade de planejamento, priorização e integração da saúde mental nos serviços de saúde pré-natal, principalmente no ambiente da Atenção Primária à Saúde, por parte de gestores de saúde e formuladores de políticas.

**Highlights:**

**(1)** Presents the risk of depression during pregnancy in usual risk antenatal care.

**(2)** Highlights the importance of screening for the risk of depression during prenatal care.

**(3)** Concludes that a higher risk of depression in pregnancy is associated with unemployment.

## Introduction

Although pregnancy is considered a normal psychosocial event for women, it can still result in major psychological changes and lead to a range of depressive behaviors among pregnant women ^(^ 1 ^)^. 

Major depressive disorder, referred to as depression in this study, is a global public health problem and a social issue characterized by a persistent and profound state of low mood^(^ 2 ^)^. Depression affects approximately 300 million individuals worldwide, making it a leading cause of disability^(^ 3 ^)^.

Depression during pregnancy, also known as prenatal depression or antepartum depression, has become a serious problem, and its long-term adverse effects have been studied and documented for a long time. Evidence confirms that depression can adversely affect both the mother and the child^(^ 4 ^)^, impact the child’s cognitive ability and development of language and behavior^(^ 5 ^)^, and serve as a predictor of postpartum depression^(^ 6 ^)^, negative neonatal outcomes^(^ 7 ^)^, adverse obstetric outcomes^(^ 8 ^)^, and negative social and personal adjustments in pregnant women^(^ 9 ^)^.

Therefore, early screening is recommended in several developed countries and depressive symptoms are evaluated throughout pregnancy. The American College of Gynecology and Obstetrics recommends that all patients be screened “at least once during the perinatal period for depression and anxiety symptoms using a standardized, validated tool”^(^ 10 ^)^. In this context, various instruments have been used to detect depression in the postpartum period, such as the Edinburgh Postnatal Depression Scale (EPDS)^(^ 11 ^)^, as well as general depression scales, such as Beck’s Depression Inventory (BDI)^(^ 12 ^)^ and the Hospital Anxiety and Depression Scale (HADS)^(^ 13 ^)^.

However, even though screening is recommended, its implementation in clinical practice falls significantly short, resulting in prenatal depression frequently going unrecognized and untreated. This occurs partly due to concerns regarding the safety of medication for pregnant women, as well as due to the similarity between depressive symptoms and signs commonly associated with pregnancy, such as mood swings, cognitive impairment, decreased energy levels, and changes in appetite, which can hinder accurate diagnosis^(^ 14 ^)^. Conversely, the issue can be attributed to the absence of screening protocols.

This is a common occurrence in several countries, including Brazil, where depression during pregnancy is often stigmatized and underdiagnosed and, consequently, it carries a heavier burden in low- and middle-income countries. Even though many women go undiagnosed, the prevalence of depression during pregnancy remains high. According to the World Health Organization (WHO), the rates of prenatal depression in low- and middle-income countries range from 12 to 42%^(^ 15 ^)^.

While a low-risk pregnancy, defined as one with clinically defined complications that pose no additional risks to the pregnancy, is socially and culturally linked to the well-being, self-fulfillment, and happiness of the woman, a pregnant woman may still be susceptible to depression or even develop the disorder^(^ 16 ^)^.

In this context, recognizing the risk of depression during pregnancy is of paramount importance, as early detection and understanding of cultural determinants can help mitigate potential consequences for women and their babies^(^ 14 ^)^. However, according to the 2020 Atlas of Mental Health, parental/maternal mental health promotion and prevention programs are found in only 29% of responding countries, making them the least commonly reported initiatives^(^ 17 ^)^.

The promotion and prevention of mental health during pregnancy are part of the specific objectives of the Global Mental Health Action Plan 2013-2020 of the World Health Organization^(^ 18 ^)^ and of the 2030 Agenda, with the 17 Sustainable Development Goals (SDGs), particularly Goal three, which addresses health and well-being. Target four of the three is to reduce premature mortality from Non-Communicable Diseases (NCDs) by one third by 2030, through prevention and treatment, as well as promote mental health and well-being^(^ 19 ^)^.

Despite these public health actions and policies that emphasize the importance of promoting and preventing women’s mental health, it is often overlooked during pregnancy, and psychiatric disorders such as depression remain invisible in prenatal care.

In view of the above, the study hypothesis assumes there is a high prevalence of depression risk during pregnancy in routine prenatal care. Therefore, due to the importance of the theme and the lack of studies of this nature aiming to identify this risk and assess the vulnerability of pregnant women in routine prenatal care, especially in Primary Health Care (PHC), the objective of this study was to identify the risk of depression during pregnancy among pregnant women undergoing routine prenatal care and the associated factors.

## Method

### Study design

This was an observational, descriptive, and correlational cross-sectional study, part of a larger project titled “Depression During Pregnancy Scale: Vulnerability of Pregnant Women to Depression Associated with the Perception of Nurses in Prenatal Care”.

### Data collection location

The study was conducted at a routine prenatal clinic in a city in the countryside of the State of São Paulo, Brazil. The clinic serves approximately 250 pregnant women *per* month and is located in a public maternity hospital that specializes in gynecology and obstetrics at a secondary level and is part of a larger university hospital complex.

### Period

Data were collected from May 4 to August 13, 2021, through visits to health centers conducted from Monday to Friday between 7 am and 12 noon. Two interviewers underwent prior training to ensure standardized procedures. Due to the COVID-19 pandemic, appropriate physical distancing measures and the use of recommended personal protective equipment (PPE) were implemented.

### Population

The study population consisted of a sample of pregnant women receiving routine prenatal care during the reference year 2020.

### Selection criteria

Pregnant women who received routine prenatal care, were aged 18 years or over, and were at any gestational age were considered eligible. The exclusion criterion was the inability to read and/or write.

### Participants

The sample consisted of 201 pregnant women. The total number of pregnant women who received low-risk prenatal care in the previous year (2020), according to data from the university hospital, which was 2327, was considered to determine the sample size. The prevalence of the risk of depression during pregnancy was assumed to be unknown due to limited research on the subject. Therefore, a conservative estimate of a 50% prevalence was used, allowing for any p-value^(^ 20 ^)^, with a confidence level of 95%, and a margin of error of 5%.

### Study variables

The dependent variable in the study was the risk of depression during pregnancy, categorized as lower risk or higher risk.

The independent variables included in the analysis were as follows: age (<20 years x 20-24 years x 25-29 years x 30-34 years x 35-39 years x 40-44 years), level of education (elementary education x secondary education x higher education), civil status (married/with partner x single/without a partner), religious belief (with a religion x without a religion), monthly family income (less than 1 minimum wage x 1 to 3 minimum wages x more than 3 minimum wages), occupation (unemployed x employed), color/race (white x black x *pardo* x yellow x indigenous), housing (non-owned property x owned property), gestational trimester (1^st^ trimester x 2^nd^ trimester x 3^rd^ trimester), number of pregnancies (primigravida x multigravida), number of deliveries (nulliparous x primiparous x multiparous), and history of miscarriage (yes x no).

### Instruments used

Data collection was conducted using an online survey platform developed on the G Suite^®^ platform. The survey was administered through the Google Forms^®^ survey management tool.

The online research page included an electronic form for data collection, as well as the Informed Consent Form (TCLE). The electronic form consisted of a characterization instrument, which included socio-demographic, economic, and obstetric questions developed by the researchers, and the scale *Escala de Risco de Depressão na Gravidez* (ERDEG) (Depression During Pregnancy Scale). This scale is an easy-to-use instrument constructed and validated in Brazil, designed to assess the risk of depression during pregnancy among pregnant women. It is a self-administered instrument with 24 questions with dichotomous answers ranging from 0 to 1. A score of 1 indicates the presence of a risk factor, while a score of 0 indicates its absence. The total score ranges from 0 to 24, with a score of 0 to 4 indicating a lower risk of developing depression during pregnancy, and a score of 5 or higher indicating a higher risk^(^ 21 ^)^.

It is important to mention that the questions on the electronic form were not obligatory, and the women had the right to choose not to answer any sensitive questions. Nevertheless, all participants chose to answer all the questions in the research.

The electronic form was pilot tested with 20 pregnant women, representing 10% of the intended sample size, who were not included in the final sample. No modifications to the wording or format of the electronic form were necessary, as it was well understood by the pregnant women during the pilot study.

### Data collection

The pregnant women were approached in the waiting room of the health center while they were waiting for their prenatal consultation and were given detailed explanations about the research and its ethical aspects. If they agreed to participate, data collection was conducted through the online research page, using a cell phone provided by the research team. The participant’s consent was obtained electronically on the online page, and a copy of the Informed Consent Form was either sent to their email or provided as a printed copy.

### Data treatment and analysis

The data analysis was conducted by exporting the data from the Google Forms^®^ survey management tool for the Microsoft Excel^®^ program, and then using the R program (R Core Team).

The chi-square test was used to assess the independence of variables, and the Fisher’s exact test was used to analyze the homogeneity of categorical variables. Categorical variables were described using absolute numbers and percentages, and continuous variables were presented with their mean and standard deviation. Finally, the variables were included in the logistic regression model after the bivariate analysis.

In the logistic regression model, the presence of multicollinearity among the parameters was assessed using the Variance Inflation Factor (VIF), with a threshold value set at five. After applying the VIF, the variables relevant for the model were selected using the stepwise procedure, considering the Akaike Information criterion. In the final model, the Odds Ratio was calculated for all variables, considering a 95% confidence interval and a significance level of 5% (α = 0.05) in all statistical tests.

### Ethical aspects

The larger study, called “Depression During Pregnancy Scale: Vulnerability of Pregnant Women to Depression Associated with the Perception of Nurses in Prenatal Care”, was approved by the Research Ethics Committee of the Ribeirão Preto College of Nursing, Universidade de São Paulo, under opinion number 4,474,220, in compliance with Resolution 466/12 of the National Health Council.

## Results

The study included 201 pregnant women receiving routine prenatal care. Most of them were young women, with a mean age of 26 years (±5.54) and age ranging from 18 to 43 years. The average family income was primarily around 2 minimum wages (±1.29), ranging from less than one minimum wage to a maximum of 10 minimum wages. The other socio-demographic characteristics revealed a predominance of mixed-race pregnant women, who were married or in a partnership, had completed secondary education, were unemployed, and lived in a non-owned property (Table 1).

**Table 1 -:** Socio-economic and obstetric characterization of participants (n=201). Ribeirão Preto, SP, Brazil, 2021

Variables	n*	%
**Age (years)**		
< 20	21	10.4
20 – 24	71	35.3
25 - 29	57	28.4
30 - 34	37	18.4
35 - 39	10	5.0
40 -44	05	2.5
**Civil status**		
Married/with a partner	131	65.2
Single/without a partner	70	34.8
**Education (level/years of education)**		
Elementary Education	51	25.4
Secondary Education	142	70.6
Higher Education	08	4.0
**Religious Belief**		
With religion	159	79.1
Without religion	42	20.9
**Occupation**		
Unemployed	122	63.7
Employed	73	36.3
**Color/race**		
Brown	93	46.3
White	70	34.8
Black	38	18.9
Yellow	00	0
Indigenous	00	0
**Housing**		
Non-owned property (rented, borrowed, invaded)	113	56.2
Owned property	88	43.8
**Monthly family income**		
< 1 minimum wage	03	1.5
1 to 3 minimum wages	181	90.0
> 3 minimum wages	16	8.5
**Trimester**		
1^st^ trimester	07	3.5
2^nd^ trimester	17	8.5
3^rd^ trimester	177	88.0
**No. of pregnancies**		
1 pregnancy (primigravida)	67	33.3
≥ 2 (multigravida)	134	66.7
**Number of births**		
0 births (nulliparous)	77	38.3
1 birth (primiparous)	72	35.8
≥ 2 deliveries (multiparous)	52	25.9
**History of miscarriage**		
Yes	41	20.3
No	160	79.7

*n = Number of participants

Regarding the current pregnancy, most pregnant women were in the third trimester, with an average gestational age of 34.22 weeks (SD±6.82), ranging from 8 to 41 weeks. As for past obstetric history, women were mostly multigravida, nulliparous and had no history of miscarriage (Table 1). The mean number of pregnancies was 2.28 pregnancies (SD±1.36), ranging from one to eight pregnancies; the number of previous deliveries ranged from zero to seven, with an average of 1.06 deliveries (SD±1.23); and the number of miscarriages ranged from zero to three, with a mean of 0.23 (SD±0.5).

The measure of the risk of depression during pregnancy, obtained through the application of the ERDEG, ranged from zero to 14 points, with an average of 6.15 (SD±2.69).

The socio-demographic, economic, and obstetric variables considered in this study did not show a statistically significant association with the occurrence of a higher risk of depression during pregnancy. All variables were included in the logistic regression model. However, only the occupation variable demonstrated a significant association with a higher risk of depression during pregnancy (p ≤ 0.05). Pregnant women without a job were twice more likely to be at a higher risk of depression during pregnancy compared to pregnant women with a job (Table 2).

**Table 2 -:** Logistic regression model: variables associated with the risk of depression during pregnancy. Ribeirão Preto, SP, Brazil, 2021

	Estimate	Standard error	P*	OR^†^
**Occupation**				
Unemployed	0.6947	0.3400	0.04	2.00
Constant	-0.1845	1.0167	0.85	

*p = p value; ^†^OR = Odds Ratio

## Discussion

A high risk of depression during pregnancy was observed in 62.2% (137) of pregnant women receiving routine prenatal care. This finding provides significant evidence that even in pregnancies without additional risks, women are susceptible to experiencing a deterioration in their psychological well-being. In another Brazilian study conducted in primary healthcare centers, 29.5% of pregnant women undergoing routine prenatal care were at moderate risk for depression^(^ 22 ^)^.

This result demonstrates the importance of investing in risk screening, as it allows understanding the vulnerability of pregnant women to mental illness and develop prevention strategies to avoid its onset and promote their mental health, especially within the primary healthcare setting. In this context, the principles of accessibility, bonding, universality, continuity of care, humanization, accountability, comprehensiveness of care, equity, and social participation, which are part of Primary Care, allow a closer relationship between pregnant women, as users of the healthcare system, and healthcare professionals, particularly nurses, enhancing prenatal care for the physical and mental health of pregnant women^(^ 23 ^)^.

Another important consideration is related to the nature of the instrument used, as the ERDEG is a self-administered tool that allows pregnant women to express their own experiences and perceptions regarding the risk of depression, with no external interference. This is significant because different assessment tools, combined with social, economic and cultural variations, can contribute to variability in results regarding prenatal depression among different populations. Previous studies have reported varying rates of depression, such as 5.4% in Egypt^(^ 14 ^)^, 14% in Nigeria^(^ 24 ^)^, 26.6% in Rwanda^(^ 25 ^)^, and 16% in Brazil^(^ 26 ^)^, showing that there is considerable heterogeneity in the rates due to differences in sample sizes, study design and assessment instrument.

The risk of depression in pregnant women on routine prenatal care may be, therefore, a more serious problem than commonly recognized and it should be considered for future research and prenatal health actions.

In the bivariate analysis, socioeconomic characteristics did not demonstrate a significant association with the risk of depression during pregnancy. However, a previous study demonstrated a relationship between lack of social support and depression among pregnant women^(^ 25 ^)^.

Obstetric characteristics also did not demonstrate any significant association. However, it is worth noting that the risk of depression was more prevalent in the first trimester of pregnancy. This finding is consistent with a study conducted in Ghana, Africa, which also found that depressive symptoms were more common in the first trimester^(^ 27 ^)^.

This observation could potentially be attributed to the data collection period, which coincided with the COVID-19 pandemic. Previous studies have reported higher rates of depression among pregnant women during the pandemic, which exacerbated psychological distress and disproportionately affected the vulnerable population of pregnant women^(^ 23 ^,^ 28 ^-^ 29 ^)^. During the pandemic, pregnant women were classified as a high-risk group and experienced a pregnancy with a lot of uncertainties regarding their health and the health of their fetus. This situation may have contributed to the elevated risk of depression since the first trimester of pregnancy. A study conducted in Turkey reported that 81.5% of pregnant women experienced depression during the pandemic^(^ 30 ^)^.

Another possible reason is the fact that the first trimester is associated with adaptation to pregnancy or onset of persistent symptoms. A longitudinal analysis is necessary to explore this aspect, as highlighted in a study carried out in South Africa, where depression was common in the first trimester, with a prevalence of 27%^(^ 31 ^)^.

In this context, the higher risk of depression in the first trimester of pregnancy reiterates the importance of universal screening, as recommended internationally by various organizations such as the American College of Obstetricians and Gynecologists^(^ 32 ^)^, the US Preventive Services Task Force^(^ 33 ^)^, and the American College of Nurse-Midwives^(^ 34 ^)^, but unfortunately not implemented in Brazil. This measure would enable early intervention to prevent the development of depressive disorders, facilitate timely referral of pregnant women to specialized mental health care if necessary, and reduce healthcare costs^(^ 35 ^)^.

Furthermore, it is important to underscore the significance of implementing strategies aimed at promoting the mental health of pregnant women and providing health education. This is because pregnancy is often held in high regard, but cultural norms, beliefs, and attitudes towards mental health, coupled with a lack of prioritization and limited mental health facilities can influence women’s help-seeking behaviors, contribute to stigma, affect the quality of healthcare provided, and impact the social inclusion of pregnant women experiencing mental distress^(^ 27 ^)^.

The multivariate analysis revealed that unemployed pregnant women had a higher risk of depression during pregnancy. This finding is supported by a study conducted in Nigeria, which showed that having a job reduced the chances of prenatal depression^(^ 25 ^)^. In contrast, a study from South Africa demonstrated that occupation does not have a significant impact on depressive symptoms in the second and third trimesters^(^ 31 ^)^.

The findings of this study emphasize the impact of the sociocultural context, particularly in developing countries like Brazil, where risk factors can be exacerbated and poverty and perinatal mental health disorders are more common.

Maternal mood disorders, including depression, have been consistently associated with socio-demographic risk factors such as low educational level and low income^(^ 36 ^)^. In the context of Brazil, depression is associated with structural and community stressors related to indicators of poverty, such as low education, insufficient income, social vulnerability, unfavorable housing conditions, and unemployment^(^ 37 ^)^.

The cross-sectional design of this study is a limitation, as it does not allow for the establishment of temporal or causal relationships between events. However, the study contributes to the advancement of scientific knowledge in the field of health and nursing by shedding light on the vulnerability of pregnant women to depression, which can be used to reduce the occurrence of prenatal depression through appropriate health interventions that include risk screening, implementation of psychological prenatal care, and promotion of mental health in primary healthcare settings.

## Conclusion

The high prevalence of risk of depression during pregnancy found in this study indicates the necessity of planning, prioritizing, and integrating mental health into prenatal health services, especially in the primary healthcare environment, by health managers and policymakers.

In this population of Brazilian women, the observed risk of depression during pregnancy should be recognized as a major public health problem, and efforts to address it should be undertaken. The implementation of screening tools for assessing the risk of depression during pregnancy can be a crucial step towards addressing the mental health needs of women undergoing routine prenatal care, particularly in the Primary Health Care setting, where their mental health is often neglected, and access to specialized mental health care can be challenging. In this context, it is important to have inter-professional and collaborative care in prenatal care, especially among nursing professional.

Implementing measures to create employment opportunities and ensure income security for pregnant women throughout pregnancy can be a successful strategy to reduce the risk of prenatal depression.
